# An analytical model to determine interseed attenuation effect in low‐dose‐rate brachytherapy

**DOI:** 10.1120/jacmp.v14i3.4226

**Published:** 2013-05-06

**Authors:** Habib Safigholi, Dariush Sardari, Somaye Karimi Jashni, Seied Rabi Mahdavi, Ali S. Meigooni

**Affiliations:** ^1^ Department of Radiation Medical Engineering, Science and Research Branch Islamic Azad University Tehran Iran; ^2^ Department of Forensic Medicine, Faculty of Medicine Tehran University of Medical Sciences Tehran Iran; ^3^ Department of Medical Physics, Faculty of Medicine Tehran University of Medical Sciences Tehran Iran; ^4^ Department of Radiation Therapy Comprehensive Cancer Center of Nevada Las Vegas NV USA

**Keywords:** LDR brachytherapy, interseed effect, ISA, TG‐43U1, Monte Carlo

## Abstract

Brachytherapy treatment planning systems (BTPS) are employing the American Association of Physicists in Medicine (AAPM) Task Group 43 (TG‐43)‐recommended dosimetric parameters of sources, which are measured in water. The majority of brachytherapy implant volumes are not homogeneous media. Particularly, an implant with multiple seeds significantly changes homogeneity of the implant volume. Heterogeneities, such as attenuation by adjacent seeds or interseed attenuation (ISA), are neglected to this day in all BTPS. The goal of this project is to determine a novel analytical method to evaluate the impact of the dose perturbations (P‐value) and/or interseed attenuation effect (ISA‐value). This method will be validated for low‐ and high‐energy brachytherapy seeds such as ${\;}^{125}I$ and ${\;}^{192}Ir$ using Monte Carlo (MC) simulation techniques. In this analytical model, determination of dose perturbation and interseed attenuation in a multisource brachytherapy implant is based on MC‐simulated 3D kernels of P‐values and ISA data for single active and single dummy configurations, arranged at different distances and orientations relative to each other. The accuracy of the final model in multisource implant configurations has been examined by a comparison of the calculated P‐values and ISA‐values with full Monte Carlo water simulations (FMCWS). This model enabled us to determine the total perturbation and ISA values for any multisource implant, and the results are in excellent agreement with the FMCWS data. The advantage of this model to FMCWS for daily clinical application is the speed of the calculations and ease of the implementation. The new perturbation and ISA formulism have shown a better accuracy for ${\;}^{192}Ir$ than ${\;}^{125}I$ due to Compton scattering and its independence of the atomic number of the chemical composition of the phantom materials. The maximum difference between the ISA model and FMCWS for all cases was less than 5%. This new model can provide inputs for brachytherapy planning software to consider the ISA effect in dose calculations based on TG‐43U1 algorithm. This approach is applicable for energy range of ${\;}^{125}I$ to ${\;}^{192}Ir$ sources.

PACS number: 87.53.Jw

## I. INTRODUCTION

Dose calculation algorithms provided by the American Association of Physicists in Medicine (AAPM) Task Group (TG‐43)^(1)^ are used in nearly all commercially available brachytherapy treatment planning systems (BTPS). In 2004 these algorithms were updated to TG‐43U1^(2)^ and then with a supplement to TG‐43U1S1^3^, ^4^ in 2010. These protocols are based on the superposition of a single source dose distribution obtained in the center of a homogenous water phantom with enough volume for radiation scattering. TG‐43 reports ignore the impact of sufficient phantom size effect,^(5)^ presence of various tissue types^6^, ^7^ which have different chemical compositions than water,^8^, ^9^ presence of applicators,^(10)^ and presence of other brachytherapy seeds^11^, ^12^ within the implanted volume. Brachytherapy seeds are normally composed of radioactive beads or rods encapsulated with metal with additional high‐z pellets as radio‐opaque markers for their identifications during postimplant CT localization. These high‐z materials in multisource brachytherapy implants will attenuate radiation passing through them and will impair predicted dose distributions calculated through a BTPS that utilizes the dosimetric parameters measured in homogeneous water phantom. The dose attenuation or perturbation of one seed by other seeds, located in the path of its photons, before reaching interaction points in the tissue is termed as “interseed attenuation” (ISA). ISA was first defined by Meigooni et al.^(12)^ as “the ratio of the dose to a point due to an implant as a whole over the sum of the individual doses due to all seeds in implant, for a multi‐seed implant configuration.” They concluded that the measurement mean values of the ISA for two‐plane implants of 3×3 seed arrays of ${\;}^{125}I$ seeds in a solid water phantom was 6% and the maximum was 12%, and concluded that these shortcomings can lead to inaccuracy in BTPS. While AAPM TG‐64^(13)^ guidelines for permanent prostate implant brachytherapy reported the inaccuracies as negligible, several studies have shown that the ISA is the difference between the full Monte Carlo (FMC) and superposition Monte Carlo (SMC) for multiple seed configurations in water.^9^, ^14^, ^15^ Mobit and Badragan^(14)^ have shown that the ISA effect in a 27 uniform spacing seeds implant of ${\;}^{125}I$ is up to 10%. Carrier et al.^(9)^ published that the FMC and SMC differences of ${\;}^{125}I$ for prostate's $D_{90}$ ranged from 5.8% to 12.8%. Chibani et al.^(15)^ reported that the missing ISA effect overestimates $D_{90}$ by 2% and 5% for ${\;}^{125}I$ and ${\;}^{103}Pd$ prostate implant, respectively. The ISA effect is dependent on seed composition,^(16)^ configuration,^12^, ^17^ seed density^(9)^ (number of seeds per unit implant volume), orientation,^(12)^ and seed‐to‐seed distances.^11^, ^14^ The active AAPM TG‐186^(8)^ has focused on investigating model‐based dose calculation (MBDC) algorithms beyond the shortcomings of TG‐43. MBDC algorithms, such as MC simulation techniques, are one approach to solving the ISA shortcoming. This approach requires voxel‐by‐voxel knowledge of seed density and composition.

This project is focused on a new approach based on an analytical model from precalculated dose perturbation kernels for a single active source by another source which is located at different distances and different orientations relative to the active source. These dose perturbation kernels are calculated using the MCNP5 MC simulation technique for different source models. The accuracy of this model has been examined for multisource implants with ${\;}^{125}I$ and ${\;}^{192}Ir$ brachytherapy sources. This model could be easily implemented in any BTPS.

## II. MATERIALS AND METHODS

### A. tG‐43u1 dose calculation

Dose calculation techniques in brachytherapy use the TG‐43^(1)^ or TG‐43U1^2^, ^3^, ^4^ parameters based on measurement or MC simulation in a homogenous water phantom. The two‐dimensional (2D) dose rate in a polar coordinate system around a sealed brachytherapy source, using line source approximation, is given by:

(1) $$D\cdot \left(r,\theta \right)=\Lambda \times S_{k}\times \frac{G_{L}\left(r,\theta \right)}{G_{L}\left(r_{0},\theta_{0}\right)}\times g_{L}\left(r\right)\times F\left(r,\theta \right)$$

where Λ is the dose rate constant $(cGy\;h^{1}U^{1}),S_{k}$ is the air‐kerma strength of the source $\left(U),G_{L}\;(r,\theta \right)$ is the geometry function where the subscript “L” indicates a line source, $g_{L}(r)$ is the radial dose function, and F(r, θ) is the 2D anisotropy function, and $\left(r_{o}=1\;cm,\theta_{o}=\pi /2\right)$ is the reference point on the transverse bisector of the source at 1 cm from its center. As shown in TG‐43 and TG‐43U1, dose rate distributions of multisource implants are calculated by superposition of the dose distributions from the individual sources.

### B. Brachytherapy seed models

#### B.1 Source of I‐125 (Model 6702)

Schematic dimensions and composition of the brachytherapy seed model 6702 by Amersham Health (Burlington, Ontario, Canada) are taken from TG‐43U1. The 6702 source consists of three resin spheres, each with a diameter of 0.600 mm, (resin density is $1.2\;g/{cm}^{3}$ and molecular composition is $C_{12}H_{18}NCl)$.^(18)^ The spheres are coated with ${\;}^{125}I$ which is assumed to have negligible thickness in this study. The spheres are encapsulated in a titanium tube with 0.050 mm thick walls and an outer diameter of 0.800 mm. End welds are 0.500 mm thick and are modeled as hemispheres on top of solid cylinders that have a 0.400 mm radius and are 0.100 mm thick.^(19)^ The overall length is 4.60 mm and the active length is 3.30 mm (calculated using the TG‐43, effective line source length with a seed spacing of 1.10 mm and N=3 sources). The average photon energy emitted by the 6702 seed is about 28.5 keV.^(1)^ The dosimetric parameters of this source are available in TG‐43U1 report. Although this source model is utilized in this project, the concept of ISA analytical model introduced in this project is applicable to other source models.

#### B.2 Ir‐192 source (Flexisource Model)

Dimensions of the ${\;}^{192}Ir$ Flexisource source are taken from the study by Granero et al.^(20)^ This source model consists of a 3.50 mm long ${\;}^{192}Ir$ active core (density 22.42 g/cm^3^) with a diameter of 0.60 mm. The active core is covered by a 0.85 mm diameter and 4.6 mm total length of AISI 304 stainless steel capsule (composition by weight: Fe 67.92%, Cr 19%, Ni 10%, Mn 2%, Si 1%, and C 0.08%, and density of $8\;g/{cm}^{3}$). The 304 stainless steel cable has been modeled as a cylinder of 5 mm length and 0.5 mm in diameter. The tip of the encapsulation is assumed to be a 0.108 mm thick conical section with a half angle of 23.6° and the radius of the face being 0.17 mm. The conical section is attached to a 0.49 mm long solid cylindrical section followed by a 3.6 mm long hollow section with an inner radius of 0.335 mm. Following the hollow section is a 0.40 mm long solid conical section with the half angle of the cone assumed to be 24°. Attached to the conical section is a 5 mm long section of AISI 304 stainless steel cable. The active length of this source is 3.50 mm. Although this source in reality is an HDR source model, it has been used here to simulate an LDR Ir‐192 source for multisource implant, not an HDR single source. The ISA model introduced here is applicable for any LDR Ir‐192 source model.

### C. Monte carlo calculations

The MC code MCNP version 5 (MCNP5) is used in this study.^(21)^ Sources (${\;}^{192}Ir$ or ${\;}^{125}I$) are simulated at the center of a water hemisphere of 40 cm radius (full scattering) made of cubes with $0.25\times 0.25\times 0.25\;{cm}^{3}$ scoring 3D voxels. Firstly, the radial dose and 2D anisotropy functions for ${\;}^{192}Ir$ and ${\;}^{125}I$ were calculated and validated with published data as recommended. Secondly, the ISA and P‐values problem were considered. The doses were estimated on a mesh with $81\times 81\;{cm}^{2}$ voxels $\left(0.25\times 0.25\times 0.25\;{cm}^{3}\right)$ of matrix for a $20\times 20\;{cm}^{2}$ plane. Each point is determined by its radial distance of “r” perpendicular to the long axis of the source and a Z‐distance parallel to the source longitudinal axis (along‐away table). Radial and Z‐distances were varied from −10 to 10 cm. The ^*^FMESH4 tally was used to score the dose distributions around the brachytherapy sources in a mesh with 3D voxels. The ^*^FMESH4 tally card allows the user to define a mesh tally superimposed over the problem geometry, and provides energy flux in $MeV/{cm}^{2}$ that can be converted to absorbed dose by applying suitable $\mu_{en}/\rho$ coefficients. The ${\;}^{192}Ir$ and ${\;}^{125}I$ gamma spectra used in this study have been obtained from the NuDat database^(22)^ and TG‐43 protocol,^(1)^ respectively. The β spectrum of the ${\;}^{192}Ir$ source has not been considered, since its contribution to the dose rate distribution for distances greater than 1 mm from the source is negligible due to the encapsulation and the plastic catheter through which the source is guided.^20^, ^23^ The density of the water used in the simulation was $0.998\;g/{cm}^{3}$ at 22°C as recommended in TG43U1.^(2)^ All simulations were done with $5\times 10^{8}$ photon histories. The MC statistical uncertainties were less than 3% for all cases.

### D. Analytical model for interseed attenuation (ISA) of a multisource implant

**Figure 1:**
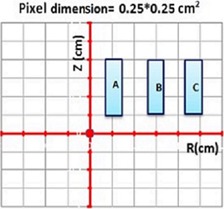
Three sources (A is active, and B and C are inactive sources) are used to derive the perturbation (P‐value) and ISA formulism (ISA‐value). These sources are places in coordinate plane of (R, Z) in away and along format. Other dimension is constant (0.25 cm) and is from −0.125 to 0.125 cm. The dose data are obtained in any voxels with dimension of $0.25\times 0.25\times 0.25\;{cm}^{3}$. The R and Z dose distances are from −10 to 10 cm for all calculation cases.

In the new analytical model, dose perturbation and ISA effect for each source in a low‐dose rate multisource implant is calculated by assuming that the source of interest is active and the other sources are inactive. The cumulative perturbations of the active source by the surrounding inactive sources were determined by multiplication of the perturbation effect from each inactive source, as shown below. The ISA effect formula used here is according to the definitions by Meigooni et al.^(12)^ and Burns and Raeside.^(11)^ In these definitions, the P‐values are defined as the ratio of the dose to a point due to an active seed, in the presence of the inactive source, to the dose without the presence of inactive source. In this project, the ISA‐value is the difference between unity and P‐value (i.e., 1‐P). In order to ease the introduction and verification of this model, three different sample implant configurations were utilized. P‐values for these samples cases were calculated using the model, as well the FMCWS of the multisource implant. For the three source arrangement (shown in Fig. 1), where the active source A has two inactive sources (B and C) located on its right, the impact of the inactive source C is calculated as:

(2) $$P_{1}=\frac{D\left(with\;C\right)}{D\left(without\;C\right)}=\frac{D\left(with\;A,\;B,\;and\;C\right)}{D\left(with\;A\;and\;B,\;without\;C\right)}$$

Similarly, the effect of the inactive source B is calculated as:

(3) $$P_{2}=\frac{D\left(with\;B\right)}{D\left(without\;B\right)}=\frac{D\left(with\;A\;and\;B,\;without\;C\right)}{D\left(with\;A\;only\right)}$$

From Eq. (3), one can extract the following equation as:

(4) $$D\left(with\;A\;and\;B,\;without\;C\right)=D\left(with\;A\;only\right)\times P_{2}$$

Place Eq. (4) on Eq. (2):

(5) $$P_{1}=\frac{D\left(with\;A,\;B,\;and\;C\right)}{D\left(with\;A\;and\;B,\;without\;C\right)}=\frac{D\left(with\;A,\;B,\;and\;C\right)}{P_{2}\times D\left(with\;A\;only\right)}$$

(6) $$P_{1}\times P_{2}=P_{MODEL}=\frac{D\left(with\;A,\;B,\;and\;C\right)}{D\left(with\;A\;only\right)}=\frac{D\left(with\;B\;and\;C\right)}{D\left(without\;B\;and\;C\right)}$$

Therefore, if we have the P‐value for the two individual inactive sources of B and C ($P_{1}$ and $P_{2}$), we can calculate the cumulative P‐value for the combination of them by multiplying the individual values. Also, ISA‐value of the combination would be $1-P_{MODEL}$. A similar concept can be proven for the total P‐value or dose deposited from one active source and N adjacent inactive sources and can be written by:

(7) $$P_{MODEL}=\left(P_{1}\times P_{2}\times \ldots \;.\times P_{N}\right)=\prod_{i=0}^{i=N}P_{i}$$

The ISA effect can be expressed by: $ISA=1-P_{MODEL}$,

(8) $$ISA_{MODEL}=[1‐(P_{1}\times P_{2}\times \ldots \;.\times P_{N})]=[(1‐\prod_{i=0}^{i=N}P_{i})]$$

With these calculation techniques, one can calculate the radial dose and anisotropy functions according to TG‐43U1 modified by the ISA effect for a single active source and adjacent dummy sources.

### E. Model validation in three simple implants

**Figure 2:**
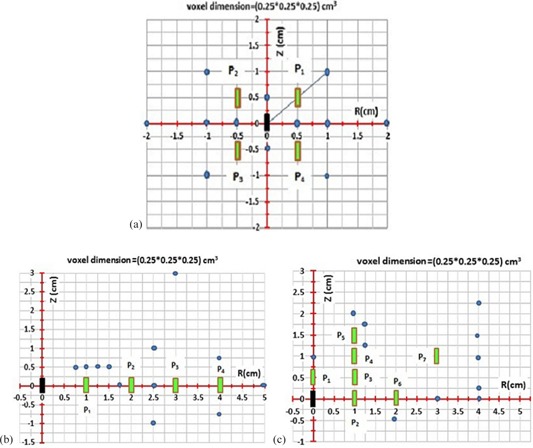
Three different simple configurations were considered to check the ISA and perturbation model accuracies ((7), (8)): (a) for case #1, the active source is in center and four other inactive seeds are at four different plans; (b) for case #2, the active source is in center and four inactive seeds are parallel to the longitudinal source axis; (c) for case #3, the active source is in center and more inactive seeds are placed along the same longitudinal axis. For all cases, the P‐values for any inactive seed position are determined with $P_{i}$ where the subscript “i” is the position number of the inactive source. The solid circles are the interest calculation points coordinates of P‐values, which are noted in 1, 2, 3, respectively.

The accuracy of the model (i.e., (7), (8)) has been examined in the energy range of ${\;}^{125}I$ to ${\;}^{192}Ir$ (28.5 keV to 380 keV) by comparison of the model base data with FMC‐simulated results for an active source being surrounded by four or more inactive sources with different configurations, as shown in Fig. 2. On this figure, the active source is shown in solid black color, while the inactive sources are shown in light green. The points of interest are shown in solid circular light blue symbols. The P‐values for any inactive seed position are determined with $P_{i}$, where the subscript “i” is the number of the inactive sources. The following sections show the details of these tests:

1. A single active source (${\;}^{125}I$ or ${\;}^{192}Ir$) in center and four inactive seeds are placed at four different quadrants relative to the active source (Fig. 2(a)). This figure shows several points of interest shown with circular symbols, located at different locations relative to the active and inactive sources. The P‐values and ISA‐values from this configuration using (7), (8) are compared with the FMC simulations for the same source arrangements.
2. A single active source is located at the origin and four inactive sources are located on one side at different distances relative to the active source, as shown in Fig. 2(b). Several points of interest are chosen at various locations relative to the active and inactive sources.
3. A single source is located at the origin and seven inactive sources distributed at different locations in one quadrant of the plane relative to the active source, as shown in Fig. 2(c). Cases 2 and 3 are used frequently for interstitial permanent prostate brachytherapy implant where the seeds are inserted parallel to the axis of needle insertion.

## III. RESULTS & DISCUSSION

### A. Monte carlo simulation validations

#### A.1 Radial dose and 2D anisotropy function

**Figure 3:**
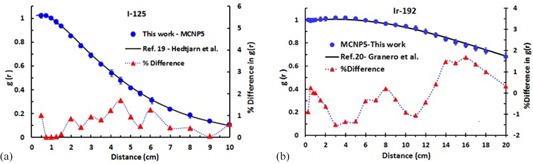
Validation of radial dose function for ${\;}^{125}I$ model 6702 (a) and ${\;}^{192}Ir$ Flexisource (b). The ${\;}^{125}I$ and ${\;}^{192}Ir$ mcg(r) data are compared with published data by Hedtjarn et al.^(19)^ and Granero et al.,^(20)^ respectively. Maximum differences between the 6702 and Flexisource references with this study are less than 1.4% and 1.8%, respectively. The MC uncertainties are at most 2.5%.

**Figure 4:**
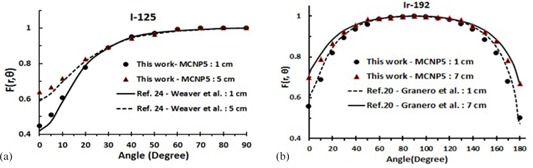
The 2D anisotropy functions (a) for ${\;}^{125}I$ are compared at 1 and 5 cm with reference published data by Weaver;^(24)^ values for ${\;}^{192}Ir$ (b) are compared with published data by Granero et al.^(20)^ Maximum differences for two seeds are limited to 2% except at small angles is reached up to 8% (TG43U1 for 6702 is reported typically 10%). The MC statistical uncertainties are at most 2.5%.

The accuracy of MC simulation in this project was validated by simulating the radial dose function, mcg(r), of ${\;}^{125}I$ (model 6702) and ${\;}^{192}Ir$ (model Flexisource) sources and comparing results with published data.^2^, ^18^, ^19^, ^20^ Figures 3(a) and (b) show comparison of the mcg(r) simulated for ${\;}^{125}I$ and ${\;}^{192}Ir$ to the published data for radial distances ranging from 0.5 to 10 cm for ${\;}^{125}I$ and 0.25 to 20 cm for ${\;}^{192}Ir$. The maximum differences between mcg(r) calculated in this project and reference data for ${\;}^{125}I$ and ${\;}^{192}Ir$ were found to be 1.4% and 1.8%, respectively. The relative MC statistical uncertainties on mcg(r) for ${\;}^{125}I$ and ${\;}^{192}Ir$ were less than 2.5% for all distances. Validation of MC simulation was also performed for the 2D anisotropy function, F(r, θ). A comparison between the present work and the published data by Weaver^(24)^ for ${\;}^{125}I$ (model 6702) and published data by Granero et al.^(20)^ for ${\;}^{192}Ir$ (Flexisource) sources are presented in Figs. 4(a) and 4(b), respectively. These results, for the radial distances of 1 and 5 cm, indicate excellent agreement (less than 2% discrepancy) with the published data by Weaver for all the angles except for small angles (less than 10°) where differences of up to 8% have been observed (TG43U1 for model 6702 is reported typically at 10%). Similarly, comparisons of ${\;}^{192}Ir$ data with the published data by Granero and colleagues are showing excellent agreement (within ±2%) for radial distances of 1 and 7 cm and at polar angles of 0°≤θ≤180° with 10° increments. The MC statistical uncertainties on F(r, θ) for the two seed models were less than 2.5%.

### B. Interseed attenuation (ISA) models

#### B.1 Perturbation values for two sources (one single active and inactive seed)

According to Eq. (7), if the P‐values are available for binary combinations of one active and one inactive source, one can calculate the P‐value for any multisource implant.

**Figure 5:**
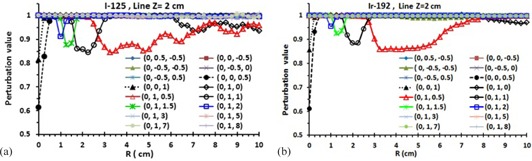
The P‐values for one active and inactive sources present for ${\;}^{125}I$ (a) and ${\;}^{192}Ir$ (b) at line Z=2cm. The positions of inactive sources are shown with different symbols. For inactive source positions at (0,0.5,−0.5),(0,0,−0.5),(0,−0.5,−0.5),(0,−0.5,0) or (0,−o.5,0.5), the corresponding P‐values in any voxel are unity. The P‐values for inactive seed positions at Z>2 cm are also unity. If the radiation path is intersected by the inactive seeds, those inactive seeds have effect on the P‐values. The P‐values statistic accuracy is better for ${\;}^{192}Ir$ than the ${\;}^{125}I$, especially for further distances due to higher photon scattering contributions. The MC uncertainties are at most 2%.

Figures 5(a) and (b), present the typical P‐value for ${\;}^{125}I$ and ${\;}^{192}Ir$ sources, respectively, assuming that the active source is located at the origin and the inactive sources are located in all quadrants of the coordinate system. These results are for points of interest located along a transverse line that is 2 cm away from the transverse bisector of the active source (i.e., Z=2 cm). These results show that, if the inactive sources are located at (0,0.5,−0.5),(0,0,−0.5),(0,−0.5,−0.5),(0,−0.5,0) or (0,−0.5,0.5), the corresponding P‐values are unity (i.e., P−Value=1.0) and hence $ISA=1-P_{value}=0$. Moreover, it has been found that there is no significant impact of the inactive sources on the dose distribution of the active source in the second, third, and fourth quadrants. In other words, the presence or absence of the dummy sources in the second, third, or fourth plan quarter of the implant have no effect on the P‐values of the first plan quarter of implant. The P‐values for dummy positions for Z>2 cm are also unity, which corresponded to dummy positions from (0, 1, 3) to (0, 1, 8) (Fig.5)). With this style, the ISA‐values are zero $\left(P_{value}=1\right)$ for any seed (active or inactive) positions after line Z=2 cm (i.e., dummy position (0, 4, 5) or (0, 8, 2.25)) and/or ISA and P‐values ≠ 0 for dummy positions before line Z=2 cm. This approach is applicable for the energy range of ${\;}^{125}I$ to ${\;}^{192}Ir$ seeds. For ${\;}^{192}Ir$, Compton scattering is important and caused the photon scattering contribution to be higher than the photoelectric absorptions for ${\;}^{125}I$. The P‐value accuracy, (3.5%), is better for ${\;}^{192}Ir$ than for ${\;}^{125}I$, especially at greater distances.

#### B.2 Full Monte Carlo water simulations (FMCWS)

To check the accuracy of the model ((7), (8)) in multiple seed implants, three FMCWS configurations (Fig. 2) were considered. 1, 2, 3 show the perturbation formulism and FMCWS data, and these are also graphically shown with varying geometry in Fig. 2. The ${\;}^{125}I$ and ${\;}^{192}Ir$ average P‐values are shown here for symmetric FMCWS for case #1 (Fig. 2(a) and Table 1) at calculation points of (0,±1,±1) are 0.8085 and 0.8675, respectively. For other calculation points in Table 1, the P‐values are approximately unity. One example is the calculation point of (0, 1, 1) for ${\;}^{192}Ir$, which yields values of $P_{2}=P_{3}=P_{4}=1$ and $P_{1}=0.867$. This confirms that the P‐values for dummies that are placed in second, third, and fourth quarters of the implant plan ($P_{2},P_{3}$, and $P_{4}$) are unity. As a rule of thumb, if a straight line is drawn from the active source to any calculation point and intersects inactive sources, those inactive sources have an effect on the perturbations, and the P‐values corresponding to other dummy sources are unity (Fig. 2(a)). On the other hand, any inactive sources which intersect the radiation path from the active source to any calculation point produce P‐values (ISA‐values) of less than one have an effect on the total perturbation (ISA). For ${\;}^{192}Ir$, the statistical accuracy is better than ${\;}^{125}I$ due to higher photon scattering. In case#1, Table 1, the maximum differences between the P‐values under FMCWS ($P_{\text{FMCWS}}$) and the perturbation model ($P_{\text{MODEL}}$) for ${\;}^{192}Ir$ and ${\;}^{125}I$ are less than 0.2% and 0.6%, respectively. 2, 3 present the P‐value calculation points equivalent to cases 2 and 3 in Figs. 2(b) and (c). For cases 2 and 3, inactive sources are parallel to each other (1 cm source‐to‐source center) and along the same longitudinal axis (0.5 cm source‐to‐source center). For cases 2 and 3, if the radiation path is intersected by any inactive sources, those inactive seeds have an effect on the P‐values and, thus, the P‐values for residual dummies are unity. For case 2 at a calculation point of (0, 5, 0), all four inactive seeds intersect the radiation path. The $P_{1},P_{2},P_{3},P_{4}$, and $P_{\text{MODEL}}$ for ${\;}^{192}Ir$ are 0.866, 0.910, 0.935, 0.946, and 0.697, respectively, while these corresponding values for ${\;}^{125}I$ are 0.890, 0.898, 0.933, 0.939, and 0.700, respectively. For a calculation point of (0, 2.5, 0), two inactive seeds intersect the radiation path. The $P_{1},P_{2},P_{3},P_{4}$, and $P_{\text{MODEL}}$ are 0.888, 0.941, 1, 1, and 0.836, respectively, while these values for ${\;}^{125}I$ are 0.855, 0.913, 1, 0.999, and 0.780, respectively. In Table 3 (case #3), the calculation points of (0, 4, 0), (0, 4, 0.25), (0, 4, 1), (0, 4, 1.5), and (0, 4, 2.25) are compared with different Z‐ axis values. According to case #3, for $P_{2}$ and $P_{6}$ at (0, 4, 0) and (0, 4, 0.25), $P_{2},P_{3}$ and $P_{7}$ at (0, 4, 1), $P_{2},P_{3}$ and $P_{7}$ at (0, 4, 1.5), and $P_{3}$ at (0, 4, 2.25), calculation points intersect the radiation path from the active source. In case #2, Table 2, the maximum differences between $P_{\text{MODEL}}$ and $P_{\text{FMCWS}}$ for ${\;}^{192}Ir$ and ${\;}^{125}I$ are less than 3.7% and 4.7%, respectively, while these values in case #3 are less than 1.2% and 4.9% for ${\;}^{192}Ir$ and ${\;}^{125}I$, respectively.

**Table 1:** Comparison between perturbation model ($P_{\text{MODEL}}$) or Eq. (7) and FMC perturbation for case #1. If a straight line from the active source to calculation points intersects the surrounding dummies, those dummies have effect on the P‐ value. This style is applicable for ${\;}^{125}I$ and ${\;}^{192}Ir$. The maximum differences between the P‐values under FMCWS ($P_{\text{FMCWS}}$) and ($P_{\text{MODEL}}$) for ${\;}^{192}Ir$ and ${\;}^{125}I$ are less than 0.2% and 0.6%, respectively. The MC statistic uncertainties are at most 2%.

*CASE #1*	*Ir‐192*		*I‐125*	
*Calculation Points*	$P_{MODEL}=P_{1}\times P_{2}\times P_{3}\times P_{4}$	$P_{FMCWS}$	$P_{MODEL}=P_{1}\times P_{2}\times P_{3}\times P_{4}$	$P_{FMCWS}$
(0, 2, 0)	0.999×1×1×1=0.999≈1	1	0.997×1×0.998×0.997=0.992≈1	0.995
(0, 1, 0)	1×0.999×0.999×1=0.998≈1	1	0.999×1×1×0.999=0.998≈1	0.997
(0, 1, 1)	0.867×1×1×1=0.867	0.867	0.810×1×1×1=0.810	0.809
(0,1,−1)	1×1×0.866×1=0.866	0.868	1×0.999×0.999×0.809=0.807	0.809
(0, 0.5, 0)	1×1×1×1=1	1	1×0.999×0.999×0.999=0.997≈1	0.999
(0,−0.5,0)	1×1×1×1=1	1	0.998×0.999×0.998×0.999=0.994≈1	0.998
(0,−1,0)	1×1×1×1=1	1	0.999×0.999×0.999×0.999=0.996≈1	0.999
(0,−2,0)	1×1×1×1=1	0.999	0.999×0.999×0.998×0.999=0.995≈1	0.997
(0, 0, 0.5)	1×1×1×1=1	1	0.999×1×0.999×1=0.998≈1	0.999
(0,0,−0.5)	1×1×1×1=1	1	0.999×0.999×0.999×0.999=0.996≈1	0.998
(0, 1, 1)	1×0.867×1×1=0.867	0.867	0.999×0.809×0.999×0.999=0.807	0.808
(0,−1,−1)	1×1×0.868×1=0.868	0.868	0.999×0.999×0.808×0.999=0.806	0.808

**Table 2:** Comparison between FMC and perturbation model ($P_{\text{MODEL}}$) for case #2. The maximum differences between $P_{\text{MODEL}}$ and $P_{\text{FMCWS}}$ for ${\;}^{192}Ir$ and ${\;}^{125}I$ are less than 3.7% and 4.7%, respectively. The MC uncertainties are at most 2.3%.

*CASE #2*	*Ir‐192*		*I‐125*	
*Calculation Points*	$P_{MODEL}=P_{1}\times P_{2}\times P_{3}\times P_{4}$	$P_{FMCWS}$	$P_{MODEL}=P_{1}\times P_{2}\times P_{3}\times P_{4}$	$P_{FMCWS}$
(0, 5, 0)	0.866×0.910×0.935×0.946=0.697	0.734	0.890×0.898×0.933×0.939=0.700	0.747
(0, 4, 0.75)	0.934×1×1×1=0.934	0.932	0.887×0.998×1×1=0.885	0.887
(0,4,−0.75)	0.935×1×1×1=0.935	0.933	0.886×0.999×0.998×1=0.883	0.886
(0, 3, 3)	0.999×0.999×0.999×1=0.997≈1	1	0.999×1×1×1=0.999≈1	0.998
(0, 2.5, 0)	0.888×0.941×1×1=0.836	0.845	0.855×0.913×1×0.999=0.780	0.789
(0, 2.5, 1)	1×1×0.999×1=0.999≈1	1	0.996×0.999×1×1=0.995	0.994
(0,2.5,−1)	1×0.999×1×1=0.999≈1	1	0.997×0.998×1×1=0.995	0.996
(0, 1.75, 0)	0.913×1×1×1=0.913	0.913	0.876×0.998×1×1=0.874	0.870
(0, 1.5, 0.5)	0.998×1×1×1=0.998≈1	0.999	0.991×1×1×1=0.991	0.992
(0, 1.25, 0.5)	1×1×1×1=1	1	0.998×1×1×1=0.998	0.996
(0, 1, 0.5)	1×1×1×1=1	1	0.998×1×1×1=0.998	0.999
(0, 0.75, 0.5)	1×1×1×1=1	1	0.999×1×1×1=0.999	0.999

**Table 3:** Comparison between FMC and perturbation model ($P_{\text{MODEL}}$) for case #3. The maximum differences between $P_{\text{MODEL}}$ and $P_{\text{FMCWS}}$ for ${\;}^{192}Ir$ and ${\;}^{125}I$ are less than 1.2% and 4.9%, respectively. The MC uncertainties are at most 2.2%.

*CASE #3*	*Ir‐192*		*I‐125*	
*Calculation Points*	$P_{MODEL}=P_{1}\times P_{2}\times P_{3}\times P_{4}\times P_{5}\times P_{6}\times P_{7}$	$P_{FMCWS}$	$P_{MODEL}\;P_{1}\times P_{2}\times P_{3}\times P_{4}\times P_{5}\times P_{6}\times P_{7}$	$P_{FMCWS}$
(0, 0, 1)	0.803×1×1×1×1×1×1=0.803	0.803	0.640×0.999×1×1×1×1×1=0.639	0.639
(0, 1, 2)	0.999×1×1×1×0.997×1×0.999=0.995	0.997	0.995×1×0.999×0.998×0.988×1×1=0.980	0.980
(0, 1.25, 1.75)	1×1×1×0.999×0.925×1×1=0.924	0.923	0.998×1×0.999×0.988×0.872×1×1=0.859	0.854
(0, 1.25, 1.25)	1×1×1×0.918×1×1×1=0.918	0.918	0.998×1×0.999×0.874×0.998×1×1=0.870	0.867
(0,2,−0.5)	1×0.986×1×1×1×1×1=0.986	0.985	1×0.997×1×1×0.999×1×1=0.996	0.999
(0, 3, 0)	1×0.876×1×1×0.999×0.935×1=0.818	0.827	1×0.859×1×0.999×0.999×0.917×0.999=0.785	0.834
(0, 4, 0)	1×0.863×1×1×0.999×0.923×1=0.796	0.808	1×0.878×0.998×0.999×1×0.905×1=0.792	0.817
(0, 4, 0.25)	1×0.863×1×1×1×0.930×1=0.803	0.815	1×0.865×0.998×1×1×0.903×0.999=0.779	0.790
(0, 4, 1)	1×0.989×0.981×1×1×1×0.987=0.958	0.960	1×0.930×0.927×0.998×1×0.999×0.964=0.829	0.829
(0, 4, 1.5)	1×0.993×0.862×0.999×1×1×0.952=0.814	0.825	1×0.980×0.866×1×1×1×0.924=0.784	0.808
(0, 4, 2.25)	1×1×0.856×1×1×0.999×1=0.855	0.854	0.998×0.999×0.863×0.998×1×0.999×1=0.858	0.850

**Figure 6:**
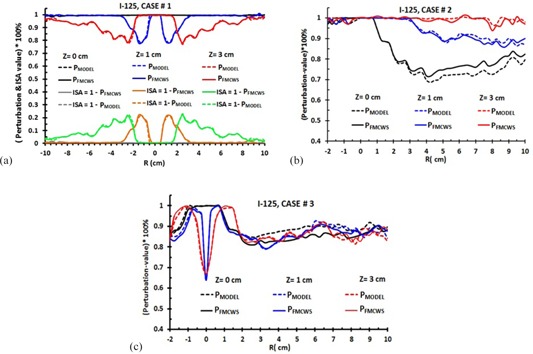
Percentage of P‐values model ((7), (8)) in comparison to FMCWS for all three simple implant geometries: (a), (b), and (c) represent data for case #1, #2, and #3, respectively. These typical data are shown for three values of Z (0, 1, and 3 cm). $P_{\text{FMCWS}}$ represent the $P_{1234}$ or $P_{1234567}$. $P_{\text{MODEL}}$ means $P_{1}\times P_{2}\times P_{3}\times P_{4}$ or $P_{1}\times P_{2}\times \ldots P_{7}$ and represent the multiplying of the P‐values from one active and inactive source. The ISA−value=1−P−value, and only graphically are shown for case #1. Maximum differences between FMCWS and MODEL are for Z=0 cm, especially in case #2 (5%). The statistic uncertainties are at most 1%.

**Figure 7:**
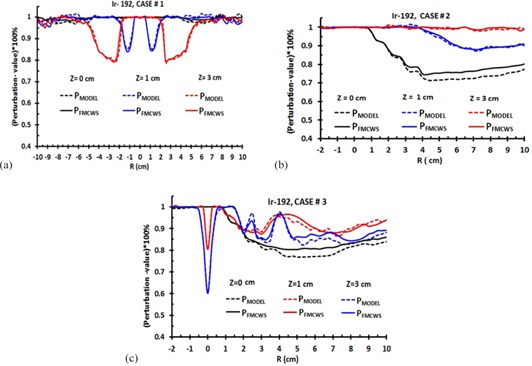
ISA‐ and P‐values percent model ((7), (8)) in comparison to FMCWS for all three ${\;}^{192}Ir$ cases. These typical data are shown for Z=0,1 and 3 cm. Figs. (a), (b), and (c) show ${\;}^{192}Ir$ data for case #1, #2, and #3, respectively. $P_{1234}$ or $P_{1234567}$ represented FMCWS. $P_{1}P_{2}P_{3}P_{4}$ or $P_{1}P_{2}P_{3}P_{4}P_{5}P_{6}P_{7}$ means $P_{1}\times P_{2}\times P_{3}\times P_{4}$ or $P_{1}\times P_{2}\times \&P_{7}$ and represent $P_{\text{MODEL}}$. The solid and dash lines are represent the $P_{\text{FMCWS}}$ and $P_{\text{MODEL}}$ values, respectively. The maximum differences between FMCWS and MODEL are at Z=0 cm, for case #2 (3.5%). The MC statistic uncertainties are at most 1.5%.

Approximately for all cases, the ISA‐values (P‐values) for ${\;}^{192}Ir$ are lower (higher) than for ${\;}^{125}I$. This is due to ${\;}^{192}Ir$ having a higher energy and dominant Compton photon scattering interaction, while in the energy range (28.5 keV) of ${\;}^{125}I$, photoelectric absorption is dominant. We select a limit such that, if the perturbation is less than 4%, we can neglect the ISA effect. If the differences between $P_{\text{FMCWS}}$ and $P_{\text{MODEL}}$ are less than 4%, their P‐ or ISA‐values are considered equal. Figures 6(a) to 6(c) show the typical ${\;}^{125}I$ data accuracy of ISA‐ and P‐values model ((7), (8)) in comparison to FMCWS for all cases. The corresponding values for ${\;}^{192}Ir$ are shown in Figs. 7(a) to 7(c). For case #1, the ISA‐ and P‐values are symmetric, and from −10 to 10 cm. The data are shown for Z=0,1, and 3 cm. The ISA‐ and P‐values at Z=0 cm are zero and unity, respectively. This is due to the inactive seed positions not being in the radiation path. For Z=1 and 3 cm, radiation paths are intersected by inactive seeds, and one can observe the perturbation effect. The maximum P‐values in case #1 for ${\;}^{125}I$ at Z=1 and 3 cm are 77.5% and 78.3%, respectively, with ISA‐values of 22.5% and 21.7%. The corresponding values for ${\;}^{192}Ir$ are 85% and 80% (P‐values) and 15% and 20% (ISA‐values), respectively. In comparison to 6, 7, one can observe that the ISA‐values for ${\;}^{125}I$ are higher than for ${\;}^{192}Ir$. In this case, the differences between FMCWS and perturbation formulism (model) are less than 0.5% and 0.8% for ${\;}^{192}Ir$ and ${\;}^{125}I$, respectively.

Data for ${\;}^{125}I$ and ${\;}^{192}Ir$ (case #2) for Z=0,1, and 3 cm are shown in 6, 7, respectively. The maximum P‐values for ${\;}^{125}I$ at Z=0,1, and 3 cm are 68%, 85%, and 94%, respectively, with ISA‐values of 32%, 15%, and 6%, respectively. The corresponding values for ${\;}^{192}Ir$ are 72%, 89%, and 100% (P‐values) and 28%, 11%, and 0% (ISA‐values), respectively. For ${\;}^{125}I$ at $Z=0\;cm,P_{1}$ and $P_{2}$ (${\text{ISA}}_{1}$ and ISA_2_) are 90% and 80% (10% and 20%), while $P_{3}$ and $P_{4}$ (${\text{ISA}}_{3}$ and ISA_4_) are 75% and 70% (25% and 30%), respectively. The ${\;}^{192}Ir$ corresponding values of $P_{1},P_{2},P_{3},P_{4}$, (or ${\text{ISA}}_{1},{\text{ISA}}_{2},{\text{ISA}}_{3},{ISA}_{4}$) are 95%, 87.5%, 80%, 75% (or 5%, 12.5%, 20%, 25%), respectively. In this case, the differences between FMCWS and the perturbation model are less than 3.5% and 5% for ${\;}^{192}Ir$ and ${\;}^{125}I$, respectively.

Data for ${\;}^{125}I$ and ${\;}^{192}Ir$ (case #3) at Z=0,1, and 3 cm, are shown in 6, 7. One can observe a maximum peak at R=0 cm for Z=1 and 3 cm. This is due to one inactive seed being placed along the longitudinal active source axis at (0, 0, 0.5). The radiation path on line Z=0 cm is not intersected by the inactive seed, so there is no peak at R=0 cm. The maximum P‐values (or ISA‐values) for ${\;}^{125}I$ at Z=0,1, and 3 cm are 80%, 67%, and 64% (or 20%, 33%, and 36%), respectively. The corresponding values for ${\;}^{192}Ir$ are 77%, 60%, and 80% (or 23%, 40%, and 20%), respectively. In case #3, the difference between FMCWS and perturbation model are less than 3% and 5% for ${\;}^{192}Ir$ and ${\;}^{125}I$, respectively. For all cases, the perturbation and ISA model ((7), (8)) are applicable from −10 to 10 cm in along and away distances and at energies of ${\;}^{125}I$ to ${\;}^{192}Ir$.

## IV. CONCLUSIONS

In this work, for first time, a new model for ISA correction based on TG‐43U1 and MC is used in dose calculation to improve the dose calculation accuracy. We provide a novel algorithm for determining the ISA model based on a group of binary combinations of one active and one inactive source using 3D MC calculations. This formulism is applicable for energies of ${\;}^{125}I$ to ${\;}^{192}Ir$ brachytherapy sources with good precision. The maximum difference between FMCWS and perturbation model for all cases are less than 3.5% and 5% for ${\;}^{192}Ir$ and ${\;}^{125}I$, respectively.

1. As a first step, MCNP5 simulations were performed to determine the dose distribution from a single active source (${\;}^{125}I$ or ${\;}^{192}Ir$) placed at the center of the full scattering spherical water phantom. The phantom is made of 3D voxels in along (z‐axis) and away (r‐axis) format to score deposited dose. The along and away distances are from −10 to 10 cm.
2. In the second step, dose perturbation has been evaluated for one single active source with an inactive source (${\;}^{125}I$ or ${\;}^{192}Ir$), located at different location and orientation relative to the active source. The maximum separation between the active and inactive sources in this study was chosen to be 6 cm to replicate the common clinical practices. The P‐values and ISA‐values are calculated in along‐away format.
3. In the third step, the accuracy of the model ((7), (8)) has been examined in the energy range of ${\;}^{125}I$ to ${\;}^{192}Ir$ (28.5 keV to 380 keV) by comparison of the model base data with FMC simulated results for an active source being surrounded by inactive sources with different configurations, as shown in Fig. 2.

Based on this approach, one could expedite the processes of dose perturbation corrections with the minimal increase in planning time as compared to the FMCWS. With this and by using step 2 above, once trained, the network generalizes to produce ISA correction response. Then for any unknown combinations of an active and inactive source for which it has not been trained, the ISA correction data will be imported to the BTPS. More detailed work using these MC dose kernel databases is currently in progress to consider the real complex multisource implant configurations and to develop software to account for the ISA missing in TG‐43‐BTPS based on Artificial Neural Network (ANN) algorithm.^(25)^ It should be noted that the geometries of the active and inactive sources were assumed to be parallel to each other.^(7)^ Variations of the relative angles between the active and inactive sources are outside of the scope of this project and could be investigated in the future.

## ACKNOWLEDGMENTS

The authors are very thankful to Drs. Shahid Awan, Hualin Zhang, and Courtney Knaup, and also Mr. Keith Sowards, for their valuable comments, sound editing analysis, and suggestions during the preparation of this manuscript.
